# The Relationship Between Intuitive Eating, Diabetes-Related Health Knowledge, and Psychological Health Outcomes in Patients with Type 2 Diabetes Mellitus

**DOI:** 10.3390/healthcare14203400

**Published:** 2026-10-12

**Authors:** Heba Mahmoud Mahmoud Mohamed, Marwa Fouad Mahmoud, Mira Naguib Abdelrazek, Medel Oabel Cabalsa, Mahmoud Abdelwahab Khedr, Mazen Baazeem, Wafa Hamad Almegewly, Rasha Fathy Dawood

**Affiliations:** 1 Department of Medical Surgical Nursing, Alexandria University, Alexandria 21519, Egypt; 2 Nursing Education Department, Faculty of Nursing, Damanhour University, Damanhour 22511, Egypt; 3 Department of Psychiatric and Mental Health Nursing, Faculty of Nursing-Alexandria University, Alexandria 21519, Egypt; mera.elfar@alexu.edu.eg or mira.elfar@riyadh.edu.sa; 4 Department of Nursing, College of Pharmacy, Nursing and Medical Sciences, Riyadh Elm University, Riyadh 11564, Saudi Arabia; 5 Leadership and Management Department, Riyadh Elm University, Riyadh 11564, Saudi Arabia; 6 College of Nursing, University of Hafr Albatin, Hafr Albatin 39921, Saudi Arabia; 7 Department of Health Information Management and Technology, College of Applied Medical Sciences, University of Hafr Albatin, Hafr Albatin 39921, Saudi Arabia; 8 Department of Community and Psychiatric Mental Health Nursing, College of Nursing, Princess Nourah Bint Abdulrahman University, P.O. Box 84428, Riyadh 11671, Saudi Arabia

**Keywords:** intuitive eating, diabetes-related health knowledge, psychological health outcomes, type 2 diabetes mellitus

## Abstract

Background: Intuitive eating, diabetes-related knowledge, and psychological well-being are relevant to person-centered diabetes care. This study examined their associations among adults with Type 2 Diabetes Mellitus (T2DM). Methods: A single-center cross-sectional study included 300 adults with T2DM recruited by convenience sampling. Intuitive eating, diabetes-related knowledge, diabetes-related distress, psychological distress, eating-related concerns, and perceived stress were assessed using self-report instruments. Associations were examined using Pearson chi-square tests and Cramér’s V. Results: Higher intuitive-eating classification was significantly associated with better diabetes-related knowledge (χ^2^(2) = 102.00, *p* < 0.001, V = 0.583), lower diabetes-related distress (χ^2^(1) = 102.80, *p* < 0.001, V = 0.585), psychological-distress category (χ^2^(3) = 25.69, *p* < 0.001, V = 0.293), lower eating-related concern category (χ^2^(2) = 95.98, *p* < 0.001, V = 0.566), and lower perceived stress (χ^2^(2) = 98.25, *p* < 0.001, V = 0.572). Conclusions: The findings indicate statistically significant cross-sectional associations but do not establish temporal direction, causality, intervention effectiveness, or metabolic safety.

## 1. Introduction

Type 2 Diabetes Mellitus (T2DM) is one of the most pressing challenges facing public health nowadays. The International Diabetes Federation (IDF) has estimated that 537 million adults are living with diabetes in 2021. It has been projected to rise of about 783 million by the year 2045, and more than 6.7 million people with ages ranging 20–79 will die from diabetes-related causes in 2021 [1]. Mostly the cause of this disease is affected by genetics and lifestyle-related factors such as poor eating habits, inadequate exercise and obesity [2]. T2DM is accompanied by several complications including CVD, retinopathy, and neuropathy leading to early deaths that cause family burden (World Health Organization) [3]. Giving more focus on other aspects, recognition of the psychological dimension has been increasing and has become the central tenet of diabetes mellitus disease management and healthy outcomes.

The growing body of evidence has shown the psychological importance of intuitive eating (IE). Since rising rates of psychological distress, anxiety and depression among T2DM patients have been reported [4], it was indicated that psychological health outcomes are significantly correlated with intuitive eating [5], while complicating dietary management and impacting suboptimal metabolic outcomes [6]. Hence, psychological health should be integrated to T2DM comprehensive management, which addresses not only the physical well-being but also the mental well-being of the patients. Traditionally, the food plans of T2DM have been the major focus of dietary management for the patient, which include caloric restriction, glycemic-index-based food pattern and structured meal planning. Although such approaches to dietary management produce improvement, challenges to psychological burden, long-term adherence to treatment regimen and their tendency to exacerbate disorders are inevitable [7,8]. With the diabetes mellitus nutrition therapy, the T2DM patient may experience less dietary self-efficacy accompanied by guilt around eating as it fosters a conflicted relationship with strict dietary guidelines [9]. Those psychological health outcomes affect T2DM patient adherence to the dietary regimen. This suggests an alternative framework for a sustainable health promoting dietary behaviors without compromising the T2DM patients’ psychological well-being.

The role of health education (HE) is a critical pillar of T2DM management. It becomes central to the concept of adoption in intuitive eating practices. It supports self-care management education and support (DSMES). The DSMES is a cornerstone of the care of T2DM patients underscoring their cognitive, psychomotor and affective abilities in managing the disease effectively [10]. The adequacy in the health information guide the T2DM patient to make informed decisions, thus, empowering them to achieve a better self-management outcome. This health education also positively influences the patient’s self-esteem, coping abilities and psychological health. The effectiveness of health education for T2DM patients, such as when it addresses the personal and psychological needs of the patients, results in a potential wherein a sustained behavior changed can be achieved [11]. Another aspect is the health literacy and cultural context of the patients, which when integrated can improve the psychological well-being of the patients. Hence, intuitive eating principles, when integrated within the T2DM management model, are a compelling and relevant approach that has received limited attention. Receiving less scholarly attention needs to be addressed to sustained effective behavior towards quality of life in diverse populations.

Although it is theoretically compelling and has empirical promise for integrating IE and HE in T2DM care, it has the presence of gaps and is still an emerging area in the extant literature that warrants further investigation. Evidently, previous research has predominantly female participants, which limits the generalizability of the study [12]. There is a lack of sustained effects on IE and HE integration to better psychological outcomes, which prevents causal inference [13]. The last issue is the poorly defined intuitive eating-based health education intervention modalities, which require careful investigation. All of these are gaps that need to be examined. Specifically, this study will examine the relationship between intuitive eating, health education, and psychological health outcomes in T2DM patients. As the study affects the quality of life of the patients, this study explores not just the association of those dimensions (intuitive eating, health education, and psychological health outcomes) but also the synergistic benefits of integrating IE and HE principles within T2DM management education programs.

## 2. Methods

### 2.1. Study Design

This descriptive cross-sectional study examined associations among intuitive eating, diabetes-related knowledge assessed using a researcher-developed questionnaire, and self-reported psychological outcomes in adults with Type 2 Diabetes Mellitus (T2DM). The observational design does not establish whether eating behaviors preceded or followed psychological symptoms or knowledge differences.

### 2.2. Study Participants

The study included 300 patients diagnosed with Type 2 Diabetes Mellitus who were recruited from the study setting according to predetermined eligibility criteria. Participants were eligible for inclusion if they had a confirmed diagnosis of Type 2 Diabetes Mellitus, were able to understand and respond to the study questionnaire, and agreed to participate voluntarily in the study. Patients were excluded if they had a medical, psychiatric, cognitive, or communication condition that could substantially interfere with their ability to understand or complete the study questionnaires.

All 300 patients approached met the inclusion criteria and agreed to participate. None had a medical, psychiatric, cognitive, or communication condition that interfered with questionnaire completion; therefore, no patients were excluded on these grounds and no refusals were recorded.

### 2.3. Study Setting and Period

The study was conducted at Damanhour Educational Hospital Diabetes Center from January 2026 to June 2026.

### 2.4. Sampling Technique

A convenience sampling technique was used to recruit 300 eligible patients with T2DM. No documented a priori power calculation was available in the study materials; therefore, the sample should be interpreted as a feasibility-based, single-center convenience sample, and this limitation is acknowledged when interpreting precision and generalizability.

During the recruitment period, 300 patients with T2DM were approached and assessed for eligibility. All 300 met the inclusion criteria, agreed to participate, and were included in the final analysis; no refusals or post-approach exclusions were documented. The resulting response rate was 100% (300/300).

### 2.5. Study Tools

Data were collected using a structured sociodemographic and clinical questionnaire, a researcher-developed 50-item questionnaire measuring diabetes-related knowledge, and standardized self-report instruments assessing intuitive eating and psychological outcomes. The researcher-developed questionnaire measures correct knowledge responses; it does not directly measure the delivery, duration, quality, or accessibility of education.

####  2.5.1. Tool I: Structured Socio-Demographic and Clinical Data Questionnaire 

A structured socio-demographic and clinical data questionnaire was developed by the researcher to collect information regarding the participants’ socio-demographic and clinical characteristics. The socio-demographic section included age, gender, marital status, educational level, and occupation, while the clinical section included previous hospitalization, duration of diabetes, regular use of controller diabetes medications, and family history of diabetes.

#### 2.5.2. Tool II: Intuitive Eating Scale-2 (IES-2)

The Intuitive Eating Scale-2 (IES-2) is a 23-item measure scored on a 1-to-5 Likert response scale (1 = strongly disagree to 5 = strongly agree). In accordance with the IES-2 scoring instructions, Items 1, 2, 3, 7, 8, 9, and 10 are reverse scored, after which all 23 items are summed and divided by 23 to obtain the overall mean IES-2 score. Higher mean scores indicate greater intuitive-eating tendencies. For descriptive interpretation in this study, the possible 1-to-5 mean-score range was prospectively divided into four equal one-point operational bands: 1.00–2.00 (low), >2.00–3.00 (moderate-low), >3.00–4.00 (moderate-high), and >4.00–5.00 (high). For the primary categorical analyses, the first two bands were combined as lower intuitive eating (mean score ≤ 3.00), whereas the latter two were combined as greater intuitive eating (mean score > 3.00). These cut-offs were study-specific operational categories rather than externally validated clinical thresholds. The dichotomization was used to support contingency-table analyses and should therefore be interpreted as an analytical classification rather than a diagnostic boundary.

The archived SPSS analytical file retains IES-2 only as the derived two-level study variable (Q11: lower intuitive-eating behaviors versus greater reliance on internal eating regulation and healthier eating attitudes). The 23 item responses and original continuous mean scores are not retained in that analytical file. Consequently, the reviewer-requested sensitivity analysis using the original continuous IES-2 score and a present-sample internal-consistency estimate cannot be reconstructed from this archive. This constraint is reported transparently and the loss of information resulting from categorization is acknowledged as an analytical limitation.

#### 2.5.3. Tool III: Health Education Questionnaire for Patients with Type 2 Diabetes Mellitus

A researcher-developed 50-item Health Education Questionnaire for Patients with Type 2 Diabetes Mellitus was used to assess diabetes-related health knowledge across eight domains: general knowledge (8 items), dietary management and nutrition (10 items), physical activity and exercise (6 items), medication adherence (6 items), blood glucose monitoring (5 items), prevention of diabetes complications (5 items), foot care (5 items), and psychological health and stress management (5 items). Each correct response was scored 1, whereas an incorrect or ‘do not know’ response was scored 0, yielding a total score of 0–50. Percentage scores were calculated from the total score. The questionnaire scoring sheet specified the following descriptive categories before analysis: poor knowledge (<50%), fair knowledge (50–74%), and good knowledge (≥75%). These categories were used to summarize knowledge level and were not interpreted as diagnostic thresholds.

#### 2.5.4. Tool IV: Problem Areas in Diabetes (PAID) Scale

The Problem Areas in Diabetes (PAID) questionnaire was used to assess diabetes-related emotional distress. The instrument contains 20 items rated from 0 (not a problem) to 4 (a serious problem). Item scores are summed and standardized to a 0–100 score by multiplying the raw total by 1.25; higher scores indicate greater diabetes-related distress. Consistent with the supplied scoring guidance, scores < 40 were categorized as low-to-moderate distress and scores ≥ 40 as high/severe diabetes-related distress.

#### 2.5.5. Tool V: Patient Health Questionnaire-4 (PHQ-4)

The Patient Health Questionnaire-4 (PHQ-4) was used as a four-item screening measure of anxiety and depressive symptoms over the previous two weeks. Each item is scored from 0 (not at all) to 3 (nearly every day), producing a total score of 0–12. Total scores were interpreted as normal (0–2), mild (3–5), moderate (6–8), and severe (9–12) psychological distress. Higher scores indicate greater psychological distress.

#### 2.5.6. Tool VI: Eating Disorder Examination Questionnaire (EDE-Q)

The Eating Disorder Examination Questionnaire version 6.0 (EDE-Q 6.0) was used as a self-report measure covering the previous 28 days. The instrument contains 28 questions, including attitudinal items scored on 0–6 response scales and behavioral-frequency questions concerning overeating, loss of control, self-induced vomiting, laxative use, and driven exercise. For the categorical analyses retained in the archived analytical dataset, the EDE-Q-derived score was summarized using three study-specific descriptive bands: <2 (minimal eating-related concerns), 2 to <4 (mild-to-moderate concerns), and ≥4 (higher eating-related concerns). These categories were used for descriptive and contingency-table analysis only and were not interpreted as diagnostic thresholds for an eating disorder. The archived dataset retains only this derived three-level variable, so the original continuous EDE-Q score cannot be reconstructed retrospectively.

#### 2.5.7. Tool VII: Perceived Stress Scale (PSS)

The 10-item Perceived Stress Scale (PSS) was used to assess perceived stress during the previous month. Items are scored from 0 (never) to 4 (very often); items 4, 5, 7, and 8 are reverse scored before summing the total. Total scores range from 0 to 40, with higher scores indicating greater perceived stress. The supplied scoring guidance classifies 0–13 as low stress, 14–26 as moderate stress, and 27–40 as high perceived stress.

Assessment outside the reported analyses (not an analyzed outcome).

### 2.6. Validity and Reliability

Content and face validity of the researcher-developed questionnaire were evaluated using expert and patient review. Content validity was assessed by a panel of five experts in medical–surgical nursing and internal medicine with diabetes-care expertise. Experts evaluated the items for relevance to the study objectives, comprehensiveness, clarity, simplicity, appropriateness of wording, redundancy, and adequacy of domain coverage. The documented mean expert rating was 3.89 out of 4.00, with overall content-validity agreement of 95%. Face validity was assessed during pilot testing in 30 patients with T2DM who reviewed clarity, simplicity, comprehensibility, and ambiguity of the items; overall face-validity agreement was 97%. Items requiring clarification were reviewed before finalization of the 50-item questionnaire. Because only aggregate validity results were documented, these findings are reported as overall agreement measures rather than as item-level I-CVI or scale-level S-CVI estimates. No exploratory or confirmatory factor analysis was performed; therefore, the available evidence supports preliminary content and face validity rather than full construct validation.

Face validity was assessed during the pilot study in 30 patients with T2DM who were comparable to the target population. Participants evaluated the questionnaire for clarity, simplicity, comprehensibility, and ambiguity of wording. Content validity was assessed by a panel of five experts in medical–surgical nursing and internal medicine with diabetes-care expertise, who reviewed item relevance, comprehensiveness, and appropriateness.

A pilot study was conducted with 30 patients with T2DM from the same setting to assess clarity, feasibility, applicability, and completion time of the 50-item questionnaire. Completion required approximately 20–25 min, and no modifications were made to the questionnaire after the pilot.

The 30 pilot participants were included in the final analytical sample of 300 because no changes to the questionnaire were required after pilot testing; their responses therefore contributed to the final analysis.

Because the 50 questionnaire items were dichotomously scored (1 = correct; 0 = incorrect/“do not know”), internal consistency was assessed using the Kuder–Richardson Formula 20 (KR-20). In the 300 study participants, the total 50-item questionnaire demonstrated good internal consistency (KR-20 = 0.842). Domain-specific KR-20 coefficients were 0.256 for general knowledge, 0.340 for dietary management, 0.398 for physical activity, −0.358 for medication adherence, 0.312 for blood glucose monitoring, 0.084 for complication prevention, 0.809 for foot care, and 0.704 for psychological health. Because several domain coefficients were low or negative, domain subscores were not interpreted as independently reliable scales; the total questionnaire score was therefore used as the principal knowledge measure.

Present-sample internal-consistency coefficients for the IES-2, PAID, PHQ-4, EDE-Q, and PSS could not be recalculated because the archived analytical dataset retains only derived categorical variables for these instruments rather than their item-level responses. This limitation is reported explicitly; the Health Education Questionnaire reliability was calculated from the available 50-item participant-level data.

The 50-item questionnaire was developed after review of diabetes self-management education and care guidance, including the American Diabetes Association Standards of Care in Diabetes (2025), ADA Standards of Care materials from 2023–2024 relevant to diabetes self-management education and support, and the Diabetes Self-Management Education and Support consensus guidance reported by Powers et al. These sources informed item generation across the eight knowledge domains.

### 2.7. Data Collection Procedure

After obtaining the necessary administrative and ethical approvals, the researcher approached eligible patients and explained the purpose and procedures of the study.

Participants who agreed to participate provided informed consent and completed the study instruments. The researcher provided clarification when necessary while avoiding any influence on participants’ responses.

The questionnaires were completed individually and were reviewed for completeness before being coded and entered for statistical analysis.

No missing values were present in the analytical dataset for the 300 participants. All 50 Health Education Questionnaire items were complete for all participants, and the categorical variables used in the reported analyses were also complete. Accordingly, no imputation was performed, and the denominator for the reported analyses was N = 300.

### 2.8. Ethical Considerations

The research approval was obtained from Research Ethical Committee at the Faculty of Nursing, Damanhour University, Egypt before the initiation of the study (Institutional Review Board approval no. IRB, 118-g dated 10 November 2025). All participants were informed about the purpose of the study, the voluntary nature of participation, and their right to withdraw from the study at any time without any consequences. Written informed consent was obtained from each participant.

Confidentiality and privacy were maintained throughout the study. The collected information was used exclusively for research purposes, and participants’ identities were not disclosed.

### 2.9. Statistical Analysis

Data were analyzed using SPSS version 29. Frequencies and percentages summarized categorical variables, while the total Health Education Questionnaire score was summarized using mean, standard deviation, and observed range. Pearson chi-square tests examined associations between intuitive-eating category and diabetes-related knowledge, diabetes-related distress, psychological distress, eating-related concerns, and perceived stress. Degrees of freedom and Cramér’s V were reported for each contingency table. All analytical variables were complete for N = 300, and no imputation was performed. As an exploratory sensitivity analysis for potential confounding, separate binary logistic-regression models examined higher versus lower intuitive-eating classification for each principal study measure while adjusting for age, sex, and educational level. Adjusted odds ratios (aORs), 95% confidence intervals, and *p*-values were reported. Diabetes duration was not included in the adjusted models because only 8 participants were in the <3-year category, producing unstable estimates; medication use and family history also showed limited variation and were not used as adjustment variables. Statistical tests were two-sided, with *p* < 0.05 considered statistically significant.

The archived analytical dataset contains categorized variables for IES-2, PAID, PHQ-4, EDE-Q, and PSS rather than their original item-level or continuous scores. Therefore, continuous-score descriptive and sensitivity analyses for these instruments could not be reconstructed retrospectively from the archived file. The categorical analyses are retained as the available prespecified analytical dataset, and the resulting loss of information and statistical power is acknowledged in the limitations.

## 3. Results

Table 1 presents the sociodemographic and clinical characteristics of the study participants. The study comprised 300 participants with diabetes. The majority were 50–60 years old (59.3%), whereas 30.7% were aged 40–50 years. Most patients were male (63.7%) and married (88.0%). More than half had a secondary level of education (52.0%), and a similar percentage were manual occupations (52.0%).

Concerning clinical characteristics, 75.3% had no previous hospitalization, while 24.7% reported previous hospitalization. Almost all patients (97.3%) had been living with diabetes for more than three years. Most patients (87.7%) reported using regular diabetes medications, and an equal percentage had a family history of diabetes (87.7%).

Table 2 shows that 63.3% of participants confirmed greater reliance on internal eating regulation and healthier eating attitudes, whereas 36.7% revealed lower levels of intuitive eating behaviors. Diabetes-related health knowledge assessment showed that 59.3% had good knowledge, 24.7% had fair knowledge, and 16.0% had poor knowledge. The total Health Education Questionnaire score averaged 36.64 ± 6.97 out of 50 (range 22–47). For diabetes-related emotional distress, 62.7% had low-to-moderate distress, whereas 37.3% denoted high diabetes-related distress. Assessment of psychological distress discovered that 60.0% had normal PHQ-4 scores, whereas 16.7% had mild distress, 6.0% had moderate distress, and 17.3% had severe psychological distress. Likewise, 60.0% of participants had minimal eating-related concerns, however 19.3% had mild-to-moderate eating disorder concerns and 20.7% verified a higher eating-related concern category. Perceived stress assessment showed that 60.0% experienced low stress, while 18.0% had moderate stress and 22.0% had high stress.

Table 3 Association Between Intuitive Eating and Diabetes-Related Health Knowledge.

A statistically significant association was reported between intuitive eating category and diabetes-related health knowledge (χ^2^(2) = 102.00, *p* < 0.001, Cramér’s V = 0.583). Participants with good knowledge were more frequently classified in the higher intuitive-eating group (154/178), whereas participants with poor knowledge were more frequently classified in the lower intuitive-eating group (36/48). This association is cross-sectional and does not establish direction or causality.

Table 4 Association Between Intuitive Eating and Diabetes-Related Distress.

There was a highly significant association between intuitive eating and diabetes-related distress (χ^2^(1) = 102.80, *p* < 0.001, Cramer’s V = 0.585), showing a strong relationship. Patients with healthier intuitive eating behaviors were greatly more likely to report low-to-moderate diabetes distress (160/188), whereas those with lower intuitive eating behaviors were more probable to experience high diabetes-related distress (82/112).

Table 5 Association Between Intuitive Eating and Psychological Distress (PHQ-4).

A statistically significant association was found between intuitive eating behaviors and psychological distress (χ^2^(3) = 25.69, *p* < 0.001, Cramer’s V = 0.293). While the association was small-to-moderate, patients with healthier intuitive eating behaviors were more probable to have normal or mild psychological distress, however severe psychological distress was more predominant among participants with lower intuitive eating behaviors.

Table 6 Association Between Intuitive Eating and Eating Disorder Psychopathology.

Intuitive eating was significantly associated with eating-related concerns (χ^2^(2) = 95.98, *p* < 0.001, Cramer’s V = 0.566), representing a strong association. Patients with healthier intuitive eating behaviors were mostly classified as having minimal eating-related concerns (154/180), although a higher eating-related concern category was significantly more common among those with minor intuitive eating behaviors (42/62).

Table 7 Association Between Intuitive Eating and Perceived Stress.

A highly significant association was found between intuitive eating behaviors and perceived stress (χ^2^(2) = 98.25, *p* < 0.001, Cramer’s V = 0.572), indicating a strong relationship. Patients with healthier intuitive eating behaviors were mainly characterized by low perceived stress (154/180), however individuals with lower intuitive eating behaviors were more likely to have moderate or high stress levels.

Exploratory adjusted logistic-regression analyses were also conducted with higher intuitive-eating classification as the dependent variable and age, sex, and educational level as covariates. The principal associations remained statistically significant at the model level. Compared with poor diabetes-related knowledge, good knowledge was associated with higher odds of being in the higher intuitive-eating group (aOR = 17.38, 95% CI 7.55–39.99, *p* < 0.001). Low-to-moderate diabetes-related distress versus high distress was associated with higher odds of higher intuitive eating (aOR = 15.41, 95% CI 8.28–28.68, *p* < 0.001). Minimal eating-related concerns versus the higher-concern category were also associated with higher intuitive eating (aOR = 11.92, 95% CI 5.83–24.36, *p* < 0.001), as was low versus high perceived stress (aOR = 9.74, 95% CI 4.90–19.36, *p* < 0.001). For PHQ-4 categories, mild versus severe distress showed aOR = 8.34 (95% CI 3.08–22.56, *p* < 0.001) and normal versus severe distress showed aOR = 3.01 (95% CI 1.52–5.95, *p* = 0.002). These adjusted analyses remain observational and do not establish direction or causality.

Overall, the descriptive and cross-tabulated data suggest associations among categorized intuitive eating, diabetes-related knowledge, and psychological outcomes. Because the inferential statistics require reconciliation against the original dataset, conclusions about statistical magnitude should remain provisional. The cross-sectional findings do not establish temporal direction or causal effects.

## 4. Discussion

The present study examined the relationship between intuitive eating and psychological health outcomes among patients with Type 2 Diabetes Mellitus (T2DM). The findings showed significant associations between intuitive eating and diabetes-related health knowledge, diabetes-related distress, general psychological distress, eating-related concerns, and perceived stress. Collectively, these results support a person-centered view of diabetes management in which eating behavior is understood not only as a means of achieving metabolic targets, but also as a psychological and behavioral process that may influence patients’ emotional adjustment and capacity for sustained self-management [6,14,15,16].

The finding that the majority of participants demonstrated healthier intuitive eating behaviors suggests that many patients with T2DM may retain or develop adaptive awareness of hunger, satiety, and food-related needs despite the continuing demands of diabetes care. Nevertheless, the presence of a substantial group with lower intuitive eating indicates that dietary monitoring, fear of glycemic consequences, weight concerns, and repeated exposure to restrictive advice may complicate patients’ relationships with food. Previous research has linked intuitive eating with greater psychological well-being, more stable eating regulation, lower disordered eating, and more favorable health-related outcomes, although the evidence among adults with T2DM remains comparatively limited [8,12,17,18,19].

The significant association between intuitive eating and diabetes-related health knowledge may indicate that diabetes-related knowledge and self-management competence enable patients to interpret nutritional guidance more flexibly and confidently. Contemporary diabetes self-management education and support emphasize collaborative goal setting, problem-solving, informed decision-making, and patient empowerment rather than the passive transfer of instructions [10,14,15]. Within this framework, well-informed patients may be better positioned to distinguish physiological hunger from emotional or externally driven eating while still considering medication schedules, carbohydrate intake, and individualized glycemic goals [20]. Because the study was cross-sectional, however, it is also possible that patients with healthier eating regulation are more receptive to education or more actively engaged in self-care.

The association between intuitive eating and diabetes-related distress is clinically important. Diabetes distress reflects the emotional burden of living with the daily demands of glucose monitoring, medication use, dietary decisions, and fear of complications, and it is conceptually distinct from major depression even though the two may overlap [1,21,22]. Greater confidence and autonomy in eating decisions may reduce feelings of failure, guilt, and being overwhelmed. Intuitive eating may therefore support emotional adaptation by decreasing conflict between prescribed dietary expectations and patients lived experiences; conversely, high diabetes distress may impair attention to internal hunger and satiety cues and encourage rigid, avoidant, or emotionally driven eating.

The relationship between intuitive eating and general psychological distress further indicates that eating behavior is closely connected with emotional health. Depression and anxiety are common concerns in diabetes care and can adversely affect motivation, treatment adherence, physical activity, and other self-management behaviors [6,13]. Evidence from systematic reviews and meta-analyses has also shown that intuitive eating is positively associated with self-esteem, body appreciation, self-compassion, and psychological well-being and negatively associated with depressive symptoms, anxiety, and maladaptive eating attitudes [12,19]. Intuitive eating should not be considered a substitute for psychological assessment or treatment, but its principles may complement mental health care by supporting emotional awareness, self-compassion, and less judgmental responses to eating experiences.

The association with eating-related concerns is particularly relevant because diabetes management can unintentionally reinforce excessive dietary restraint, preoccupation with food, and moral judgments about eating. Such patterns may increase vulnerability to binge eating, compensatory behaviors, or cycles of restriction and loss of control, particularly among psychologically vulnerable patients [9]. Reviews of intuitive eating interventions suggest potential improvements in intuitive eating, body image, quality of life, and disordered eating outcomes [22]. In patients with T2DM, however, intuitive eating must be adapted carefully: unconditional permission to eat should not be interpreted as disregard for glycemic safety, medication-related needs, or clinically indicated nutrition therapy.

The observed association between intuitive eating and perceived stress may reflect the role of flexibility and nonjudgmental awareness in coping with food-related decisions. Rigid dietary rules can create repeated experiences of success and failure, whereas a more flexible approach may reduce guilt and cognitive burden. The wider intuitive eating literature supports associations with adaptive psychological functioning and indicates that interventions can improve several psychosocial outcomes, although heterogeneity across studies and the limited number of condition-specific trials require cautious interpretation [12,18,19].

From a nursing and clinical education perspective, these cross-sectional associations raise questions for prospective study about incorporating selected intuitive-eating principles within comprehensive diabetes care. Such approaches must complement, not replace, individualized evidence-based medical nutrition therapy, medication-related meal planning, glucose monitoring, and hypoglycemia prevention. This study did not evaluate an intervention or demonstrate its clinical effectiveness.

A possible stepped-care approach for future evaluation could include individualized diabetes self-management education and support, screening for diabetes distress and problematic eating, and referral to a registered dietitian or mental health professional when indicated. This is a proposed approach rather than an intervention tested in the present study.

The present findings add to the emerging literature by highlighting the relevance of intuitive eating within a T2DM population and by examining several related psychological outcomes simultaneously. At the same time, the associations should not be interpreted as evidence that intuitive eating directly causes better psychological health. Longitudinal and intervention studies are required to determine temporal direction, identify mediating mechanisms, and establish whether improvements in intuitive eating translate into clinically meaningful changes in distress, quality of life, dietary behavior, HbA1c, and other metabolic outcomes [17,18].

## 5. Conclusions

In this single-center cross-sectional sample of adults with T2DM, categorized intuitive eating was associated with questionnaire-based measures of diabetes-related knowledge and psychological symptoms. These results cannot establish the direction or cause of the associations, demonstrate the effectiveness of intuitive-eating interventions, or confirm metabolic safety. The findings require confirmation through adequately controlled longitudinal and intervention studies after reconciliation of the statistical results.

## 6. Recommendations

Clinicians may consider individualized assessment of diabetes knowledge, psychological distress, and eating-related concerns alongside established diabetes self-management education and support. Any incorporation of intuitive-eating concepts should be coordinated with evidence-based medical nutrition therapy, prescribed treatment, and glycemic safety considerations. Prospective studies should evaluate potential benefits and harms before recommending a specific intuitive-eating intervention based on these data.

## 7. Study Limitations

Several limitations require consideration. The cross-sectional design precludes causal or temporal interpretation, and reverse or bidirectional relationships are possible. Convenience sampling from a single clinical center limits external validity. Self-report measures may introduce recall, social-desirability, and common-method biases. Dichotomization and categorization of scale scores may reduce information and statistical power. Although the total Health Education Questionnaire demonstrated good internal consistency, several domain-specific KR-20 coefficients were low or negative, and formal construct validation was not performed. Adjusted analyses were limited to age, sex, and education because some clinical covariates had sparse or limited variation, and residual confounding remains possible. Objective glycemic indicators such as HbA1c/CGM, BMI, detailed medication classes, hypoglycemia frequency, and dietary-quality measures were not available in the analytical dataset; therefore, metabolic safety or effectiveness cannot be inferred.

## Figures and Tables

**Table 1:** Sociodemographic characteristics of the study participants.

Variables	Categories	Frequency	Percent
Age	20–30	15	5.0
	30–40	15	5.0
	40–50	92	30.7
	50–60	178	59.3
Gender	Male	191	63.7
	Female	109	36.3
Marital status	Married	264	88.0
	Unmarried	36	12.0
Level of education	Highly educated	122	40.7
	Secondary	156	52.0
	Non	22	7.3
Occupation	Professional	122	40.7
	Manual	156	52.0
	Non	22	7.3
Previous hospitalization	Yes	74	24.7
	No	226	75.3
Duration of diabetes, risk factors	Less than 3	8	2.7
	More than 3	292	97.3
Regular controller medications used	Yes	263	87.7
	No	37	12.3
Family history	Yes	263	87.7
	No	37	12.3

**Table 2:** Participants scores on the study scales.

		Frequency	Percent
Intuitive Eating Scale-2 (IES-2) scores	Lower levels of intuitive eating behaviors	110	36.7
	Greater reliance on internal eating regulation and healthier eating attitudes	190	63.3
Health Education Questionnaire scores	Poor knowledge	48	16.0
	Fair knowledge	74	24.7
	Good knowledge	178	59.3
Problem Areas in Diabetes (PAID) Scale scores	High diabetes-related distress	112	37.3
	Low-to-moderate distress	188	62.7
Patient Health Questionnaire-4 (PHQ-4) scores	Severe psychological distress	52	17.3
	Moderate	18	6.0
	Mild	50	16.7
	Normal	180	60.0
Eating Disorder Examination Questionnaire (EDE-Q) scores	Clinically significant eating-related concerns	62	20.7
	Mild-to-moderate concerns	58	19.3
	Minimal eating-related concerns	180	60.0
Perceived Stress Scale (PSS)scores	High stress	66	22.0
	Moderate stress	54	18.0
	Low stress	180	60.0

**Table 3:** Association between intuitive eating and diabetes-related health knowledge.

		Lower Levels of Intuitive Eating Behaviors	Greater Reliance on Internal Eating Regulation and Healthier Eating Attitudes	Statistical Test
Health Education Questionnaire scores	Poor knowledge	36	12	χ^2^(2) = 102.00; *p* = 7.10 × 10^−23^; Cramér’s V = 0.583
	Fair knowledge	50	24	
	Good knowledge	24	154	

**Table 4:** Association between intuitive eating and diabetes-related distress.

		Lower Levels of Intuitive Eating Behaviors	Greater Reliance on Internal Eating Regulation and Healthier Eating Attitudes	Statistical Test
Problem Areas in Diabetes (PAID) Scale scores	High diabetes-related distress	82	30	χ^2^(1) = 102.80; *p* = 3.71 × 10^−24^; Cramér’s V = 0.585
	Low-to-moderate distress	28	160	

**Table 5:** Association between intuitive eating and psychological distress (PHQ-4).

		Lower Levels of Intuitive Eating Behaviors	Greater Reliance on Internal Eating Regulation and Healthier Eating Attitudes	Statistical Test
Patient Health Questionnaire-4 (PHQ-4) scores	Severe psychological distress	33	19	χ^2^(3) = 25.69; *p* = 1.11 × 10^−5^; Cramér’s V = 0.293
	Moderate	7	11	
	Mild	8	42	
	Normal	62	118	

**Table 6:** Association between intuitive eating and eating disorder psychopathology.

		Lower Levels of Intuitive Eating Behaviors	Greater Reliance On Internal Eating Regulation and Healthier Eating Attitudes	Statistical Test
Eating Disorder Examination Questionnaire (EDE-Q) scores	Higher eating-related concern category	42	20	χ^2^(2) = 95.98; *p* = 1.44 × 10^−21^; Cramér’s V = 0.566
	Mild-to-moderate concerns	42	16	
	Minimal eating-related concerns	26	154	

**Table 7:** Association between intuitive eating and perceived stress.

		Lower Levels of Intuitive Eating Behaviors	Greater Reliance on Internal Eating Regulation and Healthier Eating Attitudes	Statistical Test
Perceived Stress Scale (PSS) scores	High stress	42	24	χ^2^(2) = 98.25; *p* = 4.62 × 10^−22^; Cramér’s V = 0.572
	Moderate stress	42	12	
	Low stress	26	154	

## Data Availability

The datasets used or analyzed in this study are available from the corresponding author upon request due to privacy concerns.
